# Application of Structural and Functional Connectome Mismatch for Classification and Individualized Therapy in Alzheimer Disease

**DOI:** 10.3389/fpubh.2020.584430

**Published:** 2020-11-23

**Authors:** Huixia Ren, Jin Zhu, Xiaolin Su, Siyan Chen, Silin Zeng, Xiaoyong Lan, Liang-Yu Zou, Michael E. Sughrue, Yi Guo

**Affiliations:** ^1^Department of Neurology, The Second Clinical Medical College, Shenzhen People's Hospital, Jinan University, Shenzhen, China; ^2^The First Affiliated Hospital, Jinan University, Guangzhou, China; ^3^Department of Medical Imaging, Shenzhen People's Hospital (The Second Clinical Medical College, Jinan University; The First Affiliated Hospital, Southern University of Science and Technology), Shenzhen, China; ^4^Department of Neurology, Shenzhen People's Hospital (The Second Clinical Medical College, Jinan University; The First Affiliated Hospital, Southern University of Science and Technology), Shenzhen, China; ^5^Centre for Minimally Invasive Neurosurgery, Prince of Wales Hospital, Sydney, NSW, Australia

**Keywords:** brain connectivity, diffusion tractography imaging, Alzheimer's disease, brain parcellation, functional MRI, machine learning

## Abstract

While machine learning approaches to analyzing Alzheimer disease connectome neuroimaging data have been studied, many have limited ability to provide insight in individual patterns of disease and lack the ability to provide actionable information about where in the brain a specific patient's disease is located. We studied a cohort of patients with Alzheimer disease who underwent resting state functional magnetic resonance imaging and diffusion tractography imaging. These images were processed, and a structural and functional connectivity matrix was generated using the HCP cortical and subcortical atlas. By generating a machine learning model, individual-level structural and functional anomalies detection and characterization were explored in this study. Our study found that structural disease burden in Alzheimer's patients is mainly focused in the subcortical structures and the Default mode network (DMN). Interestingly, functional anomalies were less consistent between individuals and less common in general in these patients. More intriguing was that some structural anomalies were noted in all patients in the study, namely a reduction in fibers involving parcellations in the right anterior cingulate. Alternately, the functional consequences of connectivity loss were cortical and variable. Integrated structural/functional connectomics might provide a useful tool for assessing AD progression, while few concerns have been made for analyzing the mismatch between these two. We performed a preliminary exploration into a set of Alzheimer disease data, intending to improve a personalized approach to understanding individual connectomes in an actionable manner. Specifically, we found that there were consistent patterns of white matter fiber loss, mainly focused around the DMN and deep subcortical structures, which were present in nearly all patients with clinical AD. Functional magnetic resonance imaging shows abnormal functional connectivity different within the patients, which may be used as the individual target for further therapeutic strategies making, like non-invasive stimulation technology.

## Introduction

Alzheimer disease (AD) is characterized as the most common cause of dementia with non-stop developing progression and effective strategies, even to date. It is well-known that conventional magnetic resonance imaging (MRI) imaging provides very limited insight into dementia patients (1). While patterns of atrophy can provide some indirect diagnostic evidence for one type of degenerative disease vs. another, this is relatively limited and often can be non-specific. Furthermore, individuals can have substantial age-related atrophy and not exhibit clinical signs of dementia, again suggesting that structural brain MRI has only limited ability to diagnose, stage, or guide treatment in any meaningful way for these patients (1). Growing evidence supports the idea that AD is associated with disruptions in brain activity and networks that may target specific functionally connected neuronal networks (2, 3). These facts drive interest in more sophisticated neuroimaging, such as positron emission tomography–based studies, which are able to image the amyloid and tau proteins (4), and connectomic-based approaches, leveraging imaging studies such as functional magnetic resonance imaging (fMRI) and diffusion tractography imaging (DTI) (5). A growing number of researchers work on the development of personalized, reproducible, non-invasive, and neuroscientifically interpretable biomarkers for early diagnosis or prediction of AD even on the subjective cognition decline (SCD) stage (6–8), yet most of which is focused on the consistent abnormal connection within the multimodal imaging as the combination with DTI and fMRI (9, 10). Given the subtle and often diffuse nature of dementing disorders, machine learning–based approaches provide the most realistic method for complex imaging datasets (11, 12).

Machine learning is an application of artificial intelligence that allows computers to learn automatically and improve from experience. It is one of today's most rapidly growing technical fields (13), which performs throughout science including health care (14) such as identification and classification for diseases like AD (15–17), traffic programming (18), and marketing apps designing (19), which allows us to process large-scale, multidimensional, complex datasets in this information explosion of an era. Machine learning–based analysis of connectomic data created from neuroimaging studies in patients AD has been extensively studied in the literature (5, 9, 12, 20, 21). Most such efforts utilize a method for modeling features of either DTI and/or fMRI studies, which allow a model to differentiate between some combination of healthy controls, patients with mild cognitive impairment, and those with AD. While early identification of patients who will progress to clinical AD would provide a clinically critical patient cohort who are the best candidates for disease-modifying therapies (8), models that provide a yes vs. no answer ignore the possibility of heterogeneity of phenotypes, have limited ability to provide insight into stages of the disease, and lack the ability to provide actionable information about where in the brain a specific patient's disease is located and what specifically is happening. Treatments such as repetitive transcranial magnetic stimulation (rTMS) provide a safe and potentially useful tool that may palliate symptoms in patients even if not disease-modifying, but for which it is unclear what the appropriate target is (22).

In this pilot study, we presented a different approach using machine learning to study AD which focused on characterizing the site of a structural and functional anomaly at the single-subject level. Not only did this approach provide potentially actionable information, for therapies such as rTMS, but our data suggested that specific anomalies were remarkably consistent between individuals regardless of disease staging, which suggested that they might represent fundamental steps in early symptomatology of AD, and others became increasing less consistent which indicated the possibility of heterogeneous subgroups or stages of the disease.

## Materials and Methods

### Participants

The study included 21 patients with clinically diagnosed AD between the ages of 50 and 90 years who presented to Shenzhen's People's Hospital for evaluation and 41 healthy controls with similar age and intact cognition. All research testing was performed with the approval of the local institutional review board (Shenzhen People's Hospital Medical Ethics Committee) and with informed consent from the patient and/or designated surrogate. The research has registered in the Chinese Clinical Trial Registry (ChiCTR1800019199). The demographic characteristics of the participants are listed in Table 1.

**Table 1:** Demographic and clinical characteristics of participants.

	**Healthy control** **(*n* = 41)**	**AD (*n* = 21)**	***P***
Age (years)	70.25 (0.77)	67.43 (2.35)	0.14
Gender (% female)	22 (50%)	17 (76%)	0.001^**^
Education (years)	16.56 (0.40)	10.71 (1.02)	<0.0001^****^
Handedness (% right handed)	40 (100)	21 (100)	0.99
MMSE	29.00 (0.18)	24.29 (1.05)	0.002^**^

** means a significant difference with P = 0.001;

**** *means a significant difference with p < 0.0001*.

### Clinical and Neurocognitive Assessments

We administered the same standardized neurocognitive test to participants in both the AD and HC groups. All patients underwent standard neurologic testing in addition to the Mini-Mental Status Examination (MMSE) (23) and the Montreal Cognitive Assessment (24) to confirm the diagnosis. MMSE was used for the comparison between the AD and HC groups, based on the correction of educational level; patients were classified as cognitive decline where ≤18 MMSE. In the AD group, 17 of 21 patients were female, which had a significant difference with HC (*P* = 0.001), despite we included equal proportions of gender in HC, in clinical setting; two-thirds of persons diagnosed with AD are women. There was also a notable difference in education between two groups (*P* < 0.0001), which was consistent with the research that older adults with at least 16 years of education had less of the progressive neurodegeneration associated with AD. The MMSE in the AD group was decreased significantly compared with HC (*P* = 0.002). The participants had suffered approximately 3.2 years from AD or a noticeable cognition decline with a variation from 2 up to 10 years.

### Inclusion and Exclusion Criteria for AD

For inclusion criteria, (1) a diagnosis of probable AD according to the National Institute of Neurological and Communicative Disorders and Stroke (NINCDS) and the Alzheimer's Disease and Related Disorders Association (ADRDA) (NINCDS-ADRDA) (25), (2) at age 50 to 90 years, (3) with ≤18 MMSE score, and (4) current symptomatic treatment of AD.

And for the exclusion criteria, any other causes for cognitive decline (1) prior or current neurological or central nervous system disorders, (2) psychiatric disorder such as schizophrenia, major depression, or any other psychiatric condition, (3) abnormalities on MRI like lacunar infarcts, cerebral lesions, etc., and (4) the presence of associated disorders, immune, metabolic, or endocrine disorders and a history of cancer, etc., (5) use of prohibited medication or alcohol abuse, and (6) a diagnosis of AD and concomitant cerebrovascular disease.

### MRI Data Acquisition

For the HC group, we obtained 36 normal subject images from the Alzheimer's Disease Neuroimaging Initiative (ADNI) from the ADNI2 study collected on the Philips Achieva and GE Discovery MR 750 3.0-T MRI scanner. DTI was acquired on with 5 *b* = 0 baseline image and a *b* = 1,000 shell with 41-direction acquisition, field of view (FOV) = 350 * 350 mm, slice thickness 2.7 mm, 0-mm gap between slices with no overlap, full brain coverage, isotropic voxels, square 256 * 256 matrix.

Resting-state fMRI (rsfMRI) images were acquired on a 3.0-T MRI scanner, 3.312 × 3.312 × 3.312-mm voxels, 140 volumes/run, TR = 2,020 ms, TE = 30 ms, field of view = 224 × 224 mm, flip angle = 80°, 7-min run time.

For AD patients, Siemens Skyra 3.0-T MRI scanner was used for data acquisition; all patients underwent a pretreatment standard structural T1- and T2-weighted images, as well as diffusion-weighted image, and MR angiography to rule out secondary explanations for their clinical dementia.

DTI with the following parameters: with 10 *b* = 0 baseline image and a *b* = 1,000 shell with 64 direction acquisition, FOV = 224 * 224 mm, slice thickness 2 mm, 0-mm gap between slices with no overlap, full brain coverage, isotropic voxels, square 112 * 112 matrix.

rsfMRI was performed with the following parameters: T2-star EPI sequence, 3.5 × 3.5 × 3.5-mm voxels, 240 volumes/run, TR = 2,020 ms, TE = 30 ms, field of view = 224 × 224 mm, flip angle = 90°, 8-min run time.

To eliminate the difference made by MRI scanners in this study, a preprocessing step using tangent space normalization and whitening method was applied to correct the influence of the bias field to reduce misdiagnosis and improve the accuracy of diagnosis before segmentation or classification.

### rsfMRI Preprocessing

The rsfMRI images were processed using standard processing steps: (1) motion correction was performed on the T1 and BOLD images using a rigid body alignment; (2) slices with excess movement [defined as DVARS> 2 sigma (26) from the mean slice] were eliminated; (3) the T1 image was skull stripped using a convolutional neural net (CNN); this was inverted and aligned to the resting state bold image using a rigid alignment, which was then used as a mask to skull strip the rsfMRI image, (4) slice time correction and global intensity normalization was performed, (5) gradient distortion correction were performed using a diffeomorphic warping method which aimed to locally similarize the rsfMRI and T1 images, (6) High variance confounds were calculated using the CompCor method (27) as well as motion confounds were regressed out of the rsfMRI image, and the linear and quadratic signals were detrended, (7) spatial smoothing was performed using a 4-mm full width at half maximum Gaussian kernel. The personalized atlas created in previous steps was registered to the T1 image and localized to the gray matter regions. Thus, it was ideally positioned for extracting an average BOLD time series from all 379 areas (180 parcellations ×2 hemispheres, additionally with 19 subcortical structures), which yielded 143,641 correlations.

### Diffusion Tractography Preprocessing

The diffusion tractography (DT) images were processed using the Omniscient software, which employs a standard processing steps in the Python language (28): (1) the diffusion image was resliced to ensure isotropic voxels; (2) motion correction was performed using a rigid body alignment; (3) slices with excess movement (defined as DVARS >2 sigma from the mean slice) were eliminated; (4) the T1 image was skull stripped using a convolutional neural net (CNN); this was inverted and aligned to the DT image using a rigid alignment and then used as a mask to skull strip the DT; (5) gradient distortion correction was performed using a diffeomorphic warping method which aimed to locally similarize the DT and T1 images; (6) eddy current correction was performed; (7) fiber response function was estimated and the diffusion tensors were calculated using constrained spherical deconvolution; and (8) deterministic tractography was performed with random seeding, usually creating about 300,000 streamlines per brain.

### Machine Learning–Based Parcellation

Not only the ML has been largely used in the prediction for internet-of-Things services (29) and traffic control system (30), which also been applied to the neurological science. To create a personalized brain atlas, the structural adjacency matrix was extracted as a set of fibers running between each pair of parcellations. To minimize the effects of brain atrophy, we created a machine learning–based, subject-specific version of the HCP-MMP1 (31) atlas based on DTI structural connectivity. This was created by training a machine learning model on 200 normal adult subjects by first processing T1 and DT images as above. A HCP-MMP1 atlas in NIFTI MNI space was then warped onto each brain and the structural connectivity was calculated between every pair of this atlas and a set of ROI's containing 8 subcortical structures per hemisphere as well as the brainstem based on the streamlines, which terminated within an ROI. This step both allowed the generation of feature vectors that basically a 379 × 379 structural connectivity based adjacency matrix, and generated a centroid of the parcellation, which was utilized to constrain the voxels studied for assignment to a given parcellation to a plausible area near its typical position. These feature vectors for each region were then used as a training set and the data were modeled using the eXtreme Gradient Boosting (XGBoost) method.

This model was then applied to the new subject by first warping the HCP-MMP1 atlas to the new brain and collecting a set of feature vectors of the connectivity of each voxel (32–35). The feature vectors were then used to determine if each voxel belongs to a parcellation or region. This created a version of the HCP-MMP1 atlas with subcortical components, which was not dependent on brain shape or pathologic distortion but specific for this subject while comparable between subjects.

### Personalized Anomaly Detection

Instead of trying to fit a machine learning model to the raw data, we studied these patients on an individual level by utilizing machine learning to direct us to areas that were abnormal in AD patients compared to age-similar controls. To do this, we utilized the ADNI2 dataset to generate a training set, which was processed using the same technique. We then performed a tangent space connectivity transformation, whitening, and normalization (36) to determine the range of normal correlations for each functional connectivity and structural connectivity pair in the matrix. We then excluded the one-third of pairs in both structural and functional with the highest between subject variance in the normal cohort (37), under the hypothesis that these areas might be prone to false discovery, possible due to inter-individual variability in normal subjects. Abnormal connectivity for each connection was determined as a 3-sigma outlier for that structural or functional entry. Assignment of parcellations to various large-scale brain networks was based on several previous coordinates based meta-analyses, which have been previously published research (38–41).

**Figure 1:**
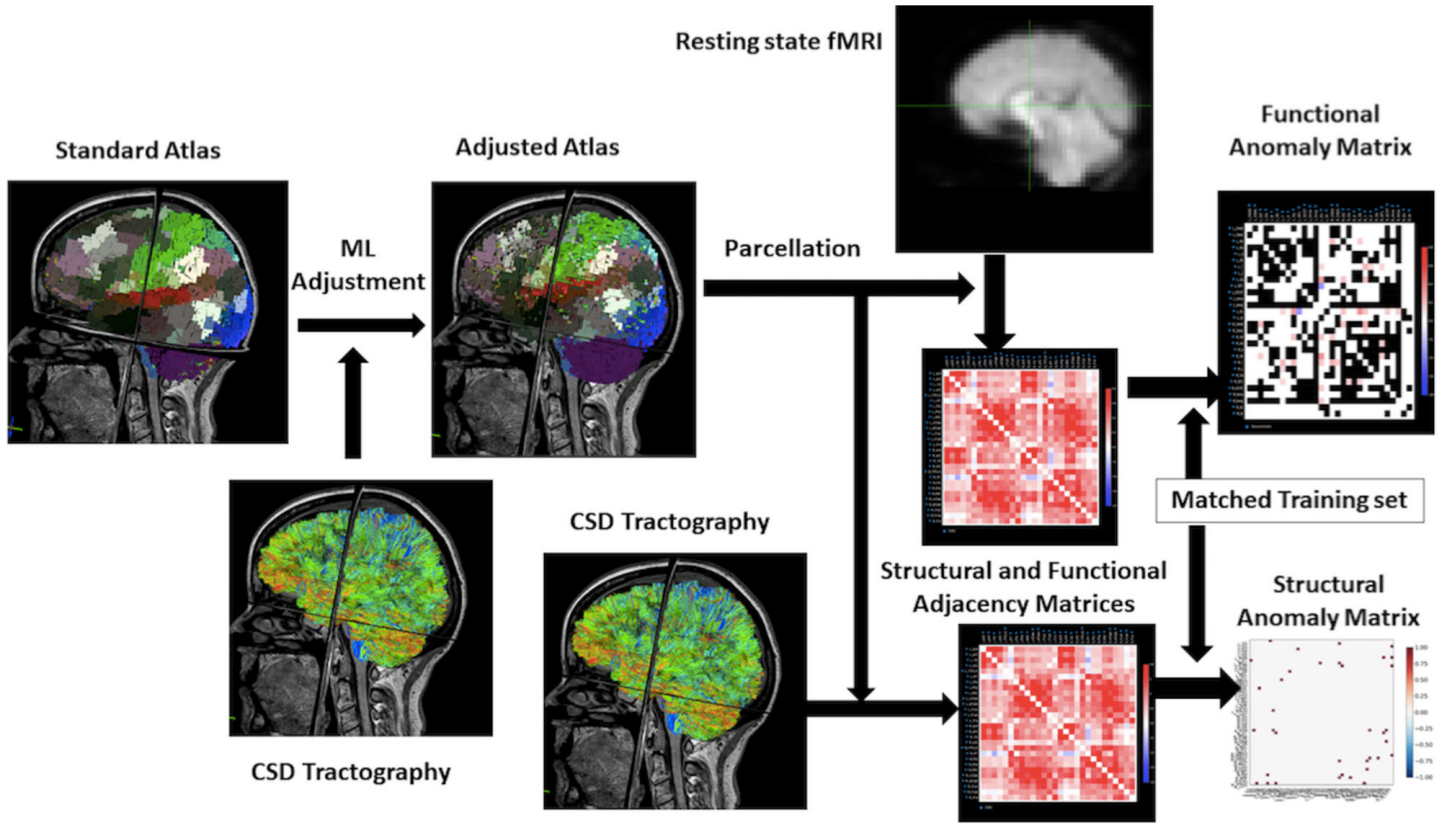
Workflow for the research. From the upper left to the right of this flowchart: the research starts with a standard atlas warped onto the brain, the boundaries are smooth because it is not machine learning–based. Then using the constrained spherical deconvolution–based tractography to adjust the atlas to personalize it. Process the rsfMRI to a functional matrix and structural MRI to a structural matrix by taking parcellation of atlas. The final step will be utilizing a training set in machine learning to make an anomaly matrix of structural and functional connectivity for further analysis.

The illustration of the data processing and model forming is shown in Figure 1.

### Statistical Analyses

All statistical analyses were conducted in SPSS software (IBM Corporation), for the comparison of demographic and clinical characteristics of participants, independent sample *T*-test analyses using two-sided tests in continuous data and a Chi-square was assessed for the discreet data.

## Results

### Anomaly Detection–Based Fingerprinting of AD-Based Anomalies

**Figure 2:**
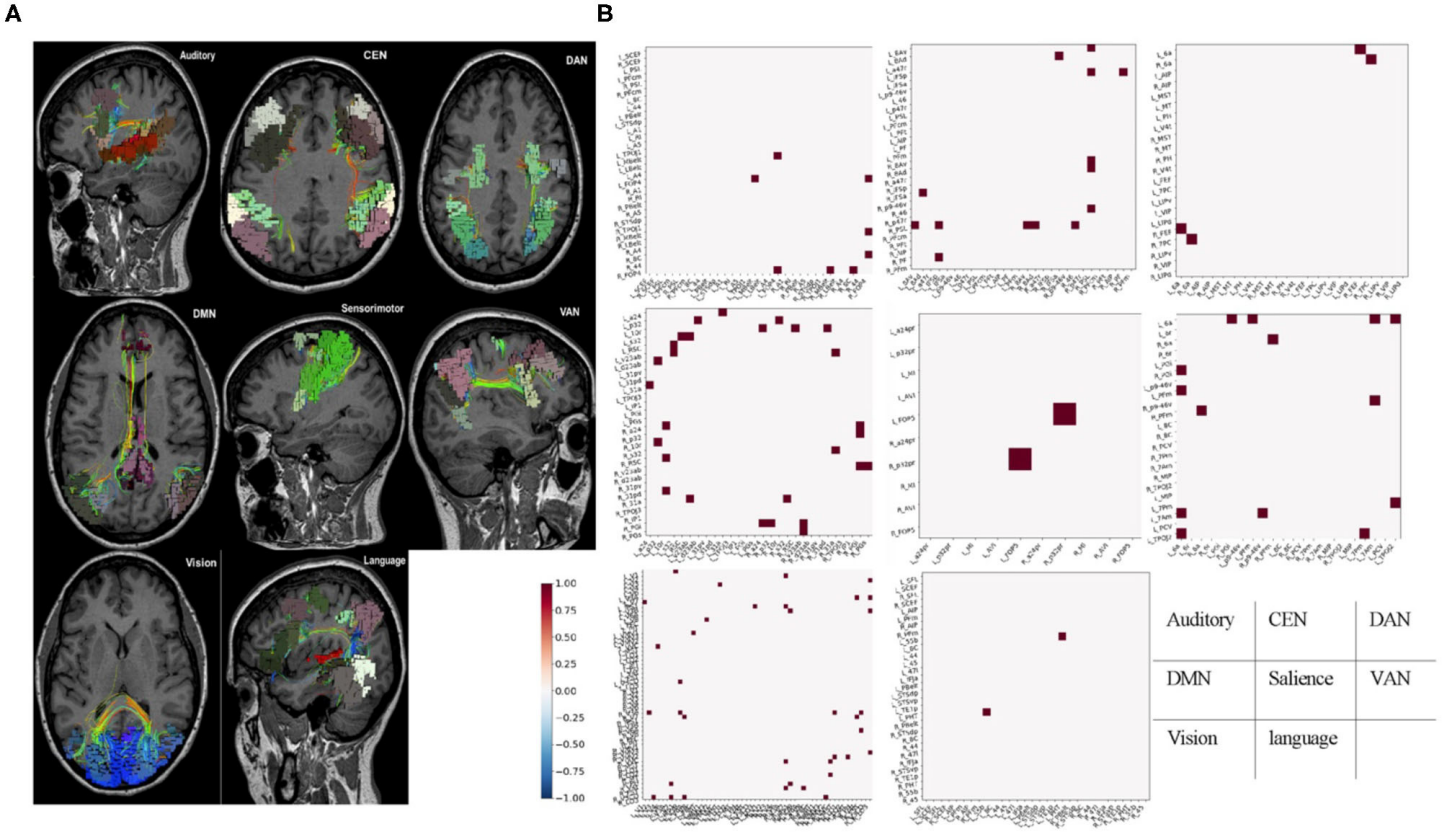
Fiber tracts and fMRI-based brain network. **(A)** Parcellations and fiber tracts–based brain network pulled out from the machine learning algorithms. Three-dimensional rendering of parcellations and tractography-based MRI images for identified set of seven canonical brain connectivity networks that Only shows tracts within areas of the network. **(B)** Example submatrices of structural anomalies for the same patient based on affiliation in the same brain-network with **(A)**. Normal or high variances (excluded areas) were indicated in white. Dots represent areas with less diffusion tractography fibers traces between them and normal, age-similar subjects. These maps provided a network-by-network fingerprint. CEN, central executive network; DAN, dorsal attention network; DMN, default mode network; VAN, ventral attention network.

Parcellations and fiber tracts–based brain network pulled out from the machine learning algorithms and an example of this matrix subset based on the affiliation of a parcellation with one of the known large-scale brain networks. This example showed the form of data these algorithms provide about specific brain networks (Figure 2). Note when we visually inspected all 21 brains, we did not note any consistent patterns between patients except that the default mode network was always abnormal in some way. It was important to note that white entries include both connections that were within normal limits compared to age-similar controls, and those connections are highly variable in the control group, suggesting that they were too interindividual variable to be meaningfully called an anomaly.

### Structural Disease Burden in AD Is Mainly in the Subcortical Structures and in DMN

To understand the behavior of data produced by our approach, we first analyzed the overall frequency of anomalies in all areas we studied to get an estimate of which areas were most frequently part of pair with a decreased number of white matter fibers on the diffusion tractography study of these patients compared to the age-similar controls. Note that two aspects of the methodology were worth reiterating. First, we parcellated the brains of both groups using a machine learning model that assigns voxels to a parcellation of subcortical structure based on which other voxels they connect to on the DTI. This means that the basic patterns of connections are held relatively consistent, and should not greatly vary due to alignment of the atlas or other similar problems. Second, while white matter connections decrease with age dependent ways, which do not necessarily cause dementia, the comparison with age-similar controls implies that this comparison should select out AD-specific connection loss.

**Figure 3:**
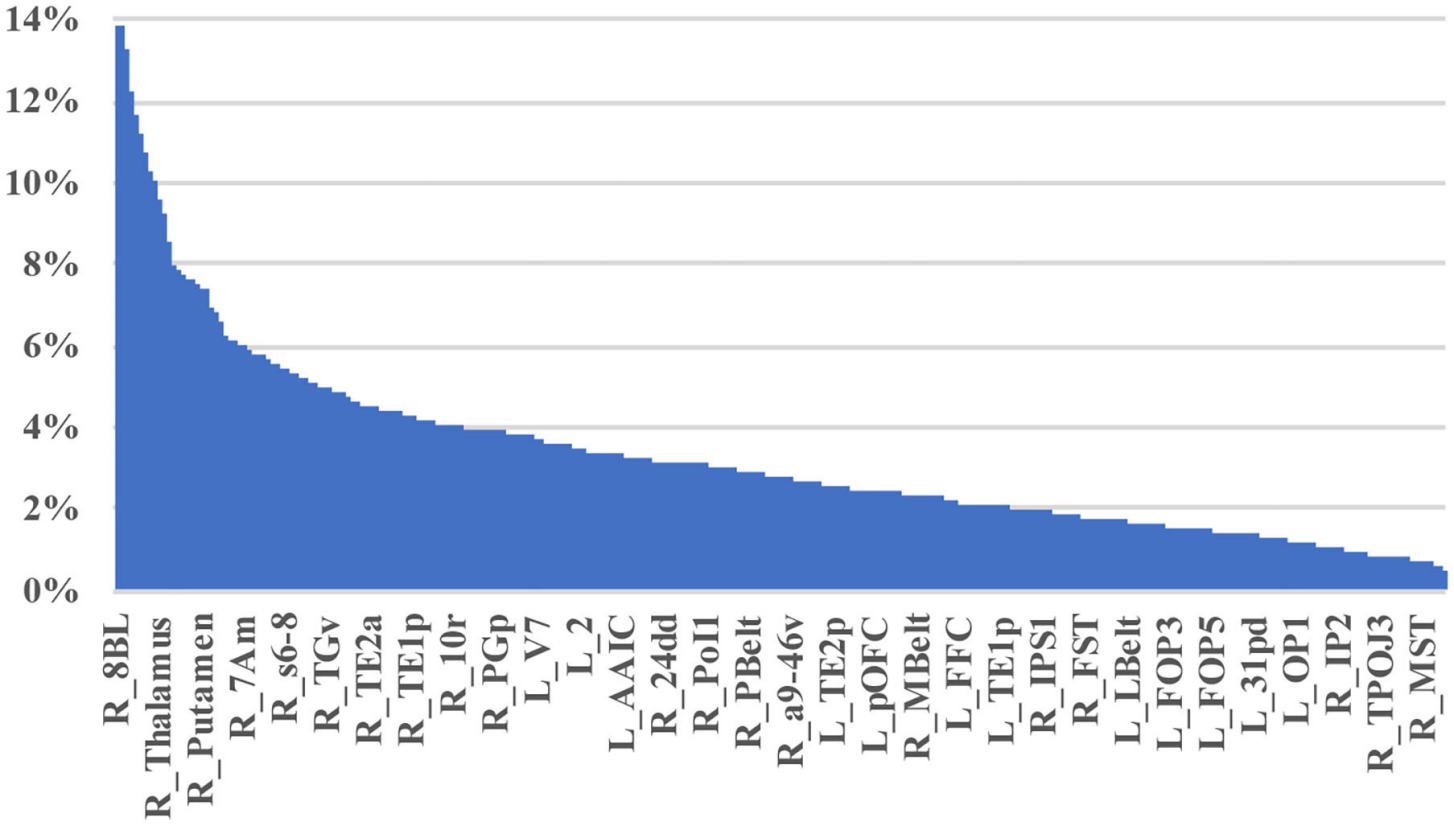
A visual depiction of structural anomaly burden in these 21 subjects. This is a set of 377 bar graphs representing the total fractions of anomalies noted in each of the cortical parcellations and subcortical regions of interest expressed as a total % of possible anomalies. This gives a sense of which connections are most consistently abnormal compared to normal age-similar but healthy controls in non-variable areas. Note there are two inflection points in this graph that demonstrate steep transitions in the data. Areas to the left of the first inflection point are mostly subcortical structures, including the putamen, caudate, and thalamus, among others, and areas 10v, right 9M, bilateral 8BM, and right area 8BL. Areas between the two inflection points mainly include regions within the anterior cluster of the Default mode network. Most other areas have a lower anomaly burden and are to the right of the second inflection point.

Table 2 demonstrates the areas with the highest fraction of their possible anomalies in all 21 patients who had an anomaly. We noted that that the top 23 areas had decreased numbers of fibers between the area and 7.6 and 13.85% of all possible target areas in all 21 patients studied (at least among the low variance options). Figure 3 shows this structural anomaly burden as a series of bar graphs. This demonstrates two natural inflection points where the burden drops, suggesting somewhat significant changes in behavior. As Table 1, shows, the majority of the high anomaly burden areas are subcortical and include basal ganglia structures, the dorsal diencephalon, and areas 8BL, and 8BM. Also notable are several parts of the anterior portion of the default mode network. Note that patients had at least one structural anomaly in every parcellation and subcortical area compared to healthy age-similar controls; these areas have the most frequent anomalies. Of note, neither hippocampus was among the most frequent sites of structural anomalies.

**Table 2:** Structural anomaly burden.

**Parcellation**	**No. of anomalies**	**No. of subjects with at least one anomaly**	**No. of low variance connections**	**Total potential anomalies**	**Percentage of total %**
R_8BL	634	21	218	4,578	13.85
L_pallidum	592	21	204	4,284	13.82
R_pallidum	694	21	249	5,229	13.27
R_ventralDC	294	21	112	2,352	12.50
R_9m	543	21	211	4,431	12.25
R_caudate	362	21	148	3,108	11.65
R_10v	714	21	302	6,342	11.26
L_ventralDC	203	21	87	1,827	11.11
Brain stem	36	21	16	336	10.71
L_putamen	225	21	104	2,184	10.30
L_thalamus	240	21	114	2,394	10.03
L_8BM	288	21	143	3,003	9.59
R_thalamus	207	21	103	2,163	9.57
R_8BM	338	21	175	3,675	9.20
L_10v	416	21	230	4,830	8.61
R_p24	560	21	333	6,993	8.01
R_OFC	462	21	276	5,796	7.97
R_cerebellum	108	21	65	1,365	7.91
R_10pp	301	21	184	3,864	7.79
R_a24	498	21	307	6,447	7.72
L_caudate	229	21	142	2,982	7.68
L_TGd	170	21	106	2,226	7.64
R_accumbens	417	21	261	5,481	7.61

### Structural–Functional Mismatch Characterizes the Anomalies in AD

Table 2 shows a similar analysis of Functional anomalies in AD. Note that the highest-burden areas are generally not subcortical regions. The default mode areas, such as p24 and 10v are on both lists as are frontal areas 8BM and 8BL. Also note that with the exception of the right hippocampus, all of the highest functional anomaly burden areas are cortical. In other words, even though the deep structures frequently show decreased numbers of white matter fibers on with different brain regions, the less commonly show observable functional connectivity disturbances with those areas.

### Disease Defining Anomalies in AD Were Structural Changes in the Right Anterior Cingulate

To see how consistent the anomalies seen in AD occurred, and specifically if there were any connection, which was usually abnormal. Table 4 demonstrates the results of this frequency analysis on the structural connectomes of these patients. Interestingly, two anomalies were seen in all 21 patients, and 3 anomalies were seen in 20/21 patients. These involved the anterior and middle cingulate gyrus on the right as one or both pairs of abnormal structural connections. As we looked through the connections of decreasing frequency, the most consistent connections were overrepresented by right-sided and DMN anomalies, consistent with many other studies.

### The Functional Consequences of Connectivity Loss Were Cortical and Variable

Table 5 demonstrates a similar analysis of the most common functional anomalies in AD patients. Two obvious differences were notable. First, functional anomalies were far less consistent with the most common anomaly in functional connectivity only occurring in 8 patients. Second, these anomalies are corticocortical or corticohippocampal, and none appear to be corticobasal or corticothalamic. Interestingly, the abnormal functional connectivity, which was common between subjects spread into numerous networks, as opposed to mainly the DMN, and it was mostly areas that were interhemispheric or not immediately adjacent to each other. The Dorsolateral prefrontal cortex (DLPFC) and dorsomedial prefrontal cortex (DMPFC) were particularly affected, with 8BM and 8BL notable inclusions.

## Discussion

The development of personalized, reproducible, non-invasive, and neuroscientifically interpretable biomarkers are urgently needed for AD precision medicine (16, 42), yet despite remarkable advances, few such biomarkers are available. Neuroimaging using DTI and fMRI in conjunction provides objective information on the structure and function that for assessing network connectivity of the brain. In this study, we performed a preliminary exploration into a set of AD data with a goal of revising a heuristic for analyzing these patients with the goal of improving a personalized approach to understanding individual connectomes in an actionable manner. Specifically, we found that there were consistent patterns of white matter fiber loss, mainly focused around the DMN and deep subcortical structures, which were present in nearly all patients with clinical AD (Tables 2, 4). Additionally, these structural anomalies were frequent, but not universal. We also found an obvious mismatch between the structural and functional anomalies in these patients, with the latter being most cortical, and mostly areas separated at long distances from each other.

**Table 3:** Functional anomaly burden.

**Parcellation**	**No. of anomalies**	**No. of subjects with at least one anomaly**	**No. of low variance connections**	**Total potential anomalies**	**Percentage of total %**
L_8BM	577	19	377	7,163	8.06
R_PFt	533	19	360	6,840	7.79
R_V1	540	19	374	7,106	7.60
L_9-46d	535	19	379	7,201	7.43
L_10v	500	19	378	7,182	6.96
R_hippocampus	453	19	370	7,030	6.44
L_AAIC	437	19	378	7,182	6.08
R_8BL	389	19	378	7,182	5.42
R_13l	384	19	374	7,106	5.40
L_IFJa	318	19	360	6,840	4.65
R_VMV3	327	19	373	7,087	4.61
L_PIT	306	19	360	6,840	4.47
R_MIP	314	19	371	7,049	4.45
R_PHT	290	19	345	6,555	4.42
L_IFJp	316	19	376	7,144	4.42
L_9p	310	19	371	7,049	4.40
R_PIT	303	19	367	6,973	4.35
L_s32	289	19	351	6,669	4.33
R_p24	304	19	374	7,106	4.28
L_PHA1	289	19	357	6,783	4.26
L_V4t	290	19	362	6,878	4.22
R_PoI2	264	19	334	6,346	4.16
R_2	282	19	359	6,821	4.13

**Table 4:** Frequency of structural anomalies.

**Patients**	**Affiliation 1**	**Parcellation 1**	**Parcellation 2**	**Affiliation 2**	**Hemisphere**	**Relationship**
21	Salience	R_a24pr	L_STSdp	Language	Bilateral	Intrahemispheric
	DMN	R_p24	R_24dd	Sensorimotor	Right	Intralobar
20	DMN	R_p24	R_p24pr	Salience	Right	Intralobar
	DMN	R_p24	R_33pr	DMN	Right	Intralobar
	DMN	R_33pr	R_24dd	Sensorimotor	Right	Intralobar
19	Basal ganglia	R_caudate	R_OFC	Orbitofrontal	Right	Corticobasal
	Basal ganglia	R_caudate	R_10v	DMN	Right	Corticobasal
	Orbitofrontal	R_OFC	R_putamen	Basal ganglia	Right	Corticobasal
17	Salience	R_a24pr	R_a24	DMN	Right	Intralobar
	DMN	R_7m	R_23d	DMN	Right	Intralobar
	Basal ganglia	R_pallidum	R_6a	Dorsal Premotor	Right	Corticobasal
	SPL	R_7Pm	R_23d	DMN	Right	Intralobar
16	Salience	R_p24pr	R_a24	DMN	Right	Intralobar
	Salience	R_p24pr	R_d32	DMN	Right	Intralobar
	DMN	R_23d	R_a24pr	Salience	Right	Intralobar
	Basal ganglia	R_pallidum	R_7PL	SPL	Right	Corticobasal
	DMN	R_10v	L_11l	Orbitofrontal	Bilateral	Intrahemispheric
	Basal ganglia	L_pallidum	R_8BL	DLPFC	Bilateral	Intrahemispheric
	Insula	L_52	L_PoI2	Insula	Left	Intralobar

The fact that DTI found white matter fiber anomalies, which were consistent between individuals, even being present in all patients, was a surprising finding, but aligns with other machine learning approaches (5) aimed at making the diagnosis of AD vs. normal, suggesting that these changes are early and disease defining. In other words, it is difficult to have clinical AD with a DMN with normal structural connectivity.

As important as this is, it implies that these problems are not useful for personalizing treatment approaches, or for staging. To that effect, the parcellations in the less common, but not rare groups e.g., being present in 50–65% of patients, seem like better candidates, as these might track the course of the disease better. Previews studies showed that the combination fMRI or/with DTI can be used for identification of the early stage of AD (9, 43) and classification from various manifestations dementia (15), while revealed only the abnormalities in large-scale network connectivity in several brain regions such as right hippocampal, left middle frontal gyrus, posterior cingulate, and middle cingulate gyrus on the right, which is consistent with the structural abnormal assessed with DTI in our study. The mismatch between structural and functional anomalies in our research was striking (Tables 2–5). It is interesting to speculate why this would be the case, but given the physical distance between areas common on this list, we suggest that loss of corticobasal and corticothalamic fibers, common in these patients, reduce the ability of these structures to facilitate communication with distant areas. It highlights the need to look at areas beyond the large-scale brain networks when we try to understand functional-phenotypic relationships.

**Table 5:** Frequency of functional anomalies.

**Patients**	**Affiliation 1**	**Parcellation 1**	**Parcellation 2**	**Affilliation 2**	**Hemisphere**	**Relationship**
8	Sensorimotor	R_2	L_IFJa	DLPFC	Bilateral	Interhemispheric
	DMN	L_10v	L_ProS	Visual	Left	Long range
	Insula	L_Pir	L_AAIC	Insula	Left	Intralobal
7	DMN	L_10v	R_PFt	Parietal	Bilateral	Interhemispheric
	DMN	L_10v	R_9-46d	DLPFC	Bilateral	Interhemispheric
	DMN	L_10v	L_AAIC	Insula	Left	Long range
	Lateral parietal	R_PFt	R_8BL	DLPFC	Right	Long range
	Lateral parietal	R_PFt	L_s32	DMPFC	Bilateral	Interhemispheric
	DLPFC	L_IFJa	R_SFL	Sensorimotor	Bilateral	Interhemispheric
	DLPFC	L_IFJa	R_s32	DMPFC	Bilateral	Interhemispheric
	Limbic	R_hippocampus	L_3b	Sensorimotor	Bilateral	Interhemispheric
	Limbic	R_hippocampus	R_13l	Orbitofrontal	Right	Long range
	DMN	L_d32	L_A1	Auditory	Left	Long range
	DMN	L_d32	L_OFC	Orbitofrontal	Left	
	Visual	L_ProS	L_8BM	DMPFC	Left	Long range
	Visual	R_V7	R_VMV1	Visual	Right	
	DLPFC	R_IFJa	L_OP2-3	Lateral parietal	Bilateral	Interhemispheric
	Orbitofrontal	L_pOFC	L_9p	DLPFC	Left	Long range
	DLPFC	L_9-46d	L_V4t	Visual	Left	Long range
6	DMPFC	L_8BM	R_hippocampus		Bilateral	Interhemispheric
	DMPFC	L_8BM	R_2	Sensorimotor	Bilateral	Interhemispheric
	DMPFC	L_8BM	R_PFcm	Lateral parietal	Bilateral	Interhemispheric
	DMPFC	L_8BM	R_V7	Visual	Bilateral	Interhemispheric
	DMPFC	L_8BM	R_V1	Visual	Bilateral	Interhemispheric
	DMPFC	L_8BM	L_s32	DMPFC	Left	
	DMPFC	L_8BM	R_10v	DMN	Bilateral	Interhemispheric
	DMPFC	L_8BM	L_9-46d	DLPFC	Left	
	Lateral parietal	R_PFt	R_V3A	Visual	Right	Long range
	Lateral parietal	R_PFt	R_V7	Visual	Right	Long range
	Lateral parietal	R_PFt	L_ProS	Visual	Bilateral	Interhemispheric
	Lateral parietal	R_PFt	L_31pd	DMN	Bilateral	Interhemispheric

It was well-known that DMN was considered as the most affected network in neurological and neuropsychiatric disorders, including AD, which shows a high level of activity during rest while deactivates its performance during cognitive tasks (44). These areas include the precuneus/posterior cingulate cortex, medial prefrontal cortex (MPFC), and medial, lateral, and inferior parietal cortex, and its activity holds potential as a non-invasive biomarker of incipient AD (45). Researchers have demonstrated the disconnection or decreased functional connectivity within/between DMN and other networks, which contribute to a cognition decline (46).

Regardless of the mechanism, functional data seems less consistent than structural data most in the DMN. There are good and bad points to using these data. This suggests that using machine learning–based on the variability of functional connectivity to classify or identify patients in early-stage disease, or to stage the extent of the disease, seems less promising than structural data as the anomalies seem to be more individual specific. However, the inherent variability of functional anomaly data in our patients suggests that it is highly promising at personalizing approaches to therapy, such as TMS (22). In this paradigm, an integrated understanding of the structural defects unique to that patient, as well as the functional consequences, can provide a unique approach to why certain symptoms occur in a specific patient. In other words, things that do not vary seldom provide variable outcomes.

The following are a few notes about our data modeling approach. First, parcellating the brain of structurally abnormal patients has long been a source of variability in the data, especially in the presence of brain atrophy. By using a machine learning approach based on structural connectivity patterns, we hold at least one variable (voxel identity in a parcellation) relatively constant, as the connectivity pattern should remain similar for a parcellation across brains (41, 47–49). Further, while the connectome has seemingly infinite interindividual variability, we hypothesize that clinically relevant phenotypes we are interested in at this early stage are less likely to result from the loss of rare individual variants in connectivity, and instead result from more constant interindividual connections. Thus, we eliminated many of the higher variance connectivity edges on the graphs to focus on similarities across individuals, and reduce the false discovery rate when scaling the results of machine learning models to individuals. In other words, we focused on brain connectivity, which we can more convincingly expect to be in a specific range.

As the potential treatment that non-invasively applying on cognitive decline, TMS may also begin to address etiological or syndrome's heterogeneity by targeting specific circuits to treat specific symptom clusters. However, it remains unknown whether the stimulation of different circuits is associated with improvement in different cognitive symptoms. In clinical practice, TMS targeting is usually based on scalp measurements and mostly without a flexible tracking device to fix the coil, resulting in different patients, or even the same patient during their series of sessions receiving stimulation of different sites in the prefrontal cortex.

Although there are important discoveries revealed by our study, there are also limitations. First, we included only 21 AD patients, which may lead to some potential bias for machine learning calculation-based results. Second, the way we eliminated one-third of parcellation pairs with the highest variance in the cohort of normal subjects, may have lost some original information, While, these areas were the smaller parcellations and is mainly aimed to reduce the problem of multiple comparisons (50). This should not be expected to introduce any subjective bias as it was based on the data. Finally, even after excluding one-third of the connectivity differences, the abnormities results we made have not been applied to selecting the individual target for rTMS treatment, although there may be a long way from being employed to the clinic, the outcome that we made may provide evidence for individualized and precise treatment for AD.

In conclusion, we demonstrated a machine learning–based approach to studying individual connectomes in a non-group averaged way. This critical exploratory work lays the groundwork for future larger-scale work in these patients. Our findings highlight the potential for a reproducible and generalizable functional brain imaging biomarker to aid the early diagnosis of AD and track its progression. This data-driven approach for identifying connectivity-specific targets may prove useful for other disorders and facilitate personalized neuromodulation therapy like rTMS. Collectively, our findings highlight the potential for mismatching between structural and functional brain imaging to provide a generalizable, and neuroscientifically interpretable imaging biomarker that may support clinicians in the non-invasive personalized treatment of AD. Further, our study may shed light on exploring new mechanisms and individualized stratagem based on the functional connectivity of brain networks in patients with dementia or even other neurodegenerative diseases.

## Data Availability Statement

The raw data supporting the conclusions of this article will be made available by the authors, without undue reservation.

## Ethics Statement

The studies involving human participants were reviewed and approved by Shenzhen People's Hospital Medical Ethics Committee. The patients/participants provided their written informed consent to participate in this study.

## Author Contributions

HR contributed to manuscript drafting and revising, data analysis and picture preparation. JZ and SC contributed to data acquisition and preparation. XS, SZ, XL, and L-YZ contributed to manuscript revising and data analysis. MS and YG contributed to study design, manuscript revising, data analysis, and study supervision. All authors contributed to the article and approved the submitted version.

## Conflict of Interest

The authors declare that the research was conducted in the absence of any commercial or financial relationships that could be construed as a potential conflict of interest.
